# Small Extracellular Vesicles Derived from NF2-Associated Schwannoma Cells Modulate Tumor Progression and Immunity via HSP90

**DOI:** 10.3390/curroncol32100569

**Published:** 2025-10-13

**Authors:** Ying Wang, Yuan Ren, Qi Zhang, Chao Zhang, Minjun Yan, Xin Ma, Bo Wang, Peng Li, Pinan Liu

**Affiliations:** 1 Department of Neural Reconstruction, Beijing Neurosurgical Institute, Beijing Tiantan Hospital, Capital Medical University, Beijing 100070, China; yingwang@ccmu.edu.cn; 2 Department of Neurosurgery, Beijing Tsinghua ChangGung Hospital, School of Clinical Medicine, Tsinghua University, Beijing 102218, China; rya01982@btch.edu.cn; 3 Department of Ultrastructural Pathology, The Neuropathology Center, Beijing Neurosurgical Institute, Beijing Tiantan Hospital, Capital Medical University, Beijing 100070, China; zhangqi5706@mail.ccmu.edu.cn; 4 Department of Neurosurgery, Beijing Tiantan Hospital, Capital Medical University, Beijing 100070, China; kepler001@126.com (C.Z.); shenwaiymj@mail.ccmu.edu.cn (M.Y.); xinma@mail.ccmu.edu.cn (X.M.); bowang@ccmu.edu.cn (B.W.)

**Keywords:** NF2-related schwannomatosis (NF2-SWN), exosome, MDSC, HSP90, AKT

## Abstract

**Simple Summary:**

Small extracellular vesicles (sEVs) derived from NF2-associated schwannomas (NF2-EVs) express CD9 and CD81, with a size distribution ranging from 50 to 200 nm. NF2-EVs can induce the phenotypic transformation of CD14^+^ monocytes into MDSC cells. NF2-EVs-induced MDSC cells show increased expression of *ARG1*, *iNOS*, and *BAFF*, along with heightened secretion of IL-10, TNFα, MIP-1α, G-CSF, IL-8, and IL-6. Simultaneously, they produce a large amount of ROS and are able to inhibit the proliferation of autologous T cells. Additionally, NF2-EVs promote the proliferation of NF2 schwannoma cells. NF2-EVs highly express HSP90 and can regulate AKT/p-AKT and ERK/p-ERK through HSP90, thereby affecting cell proliferation and promoting the conversion of monocytes to MDSCs, which mediates immune suppression.

**Abstract:**

In-depth exploration of tumor immune suppression mechanisms may provide new therapeutic options for NF2-associated tumors. In this study, we found that sEVs secreted by NF2-associated schwannomas (NF2-EVs) facilitate the conversion of CD14^+^ monocytes into an MDSC-like phenotype, showcasing MDSC-like inhibitory functions. Moreover, these NF2-EVs are capable of enhancing tumor cell proliferation. Through proteomic analysis and subsequent validation of the NF2-EVs, we identified elevated levels of HSP90. When we knocked down HSP90 expression in tumor cells, the sEVs secreted showed diminished capacity to convert monocytes into MDSCs and a reduced ability to promote tumor cell proliferation. Conversely, sEVs secreted by tumor cells that overexpress HSP90 displayed the opposite effects. Further mechanistic studies revealed that HSP90 could influence the expression of AKT/p-AKT and ERK/p-ERK. Our results suggest that NF2 tumor cells could regulate the AKT/p-AKT and ERK/p-ERK pathways to promote tumor cell proliferation and the formation of an immunosuppressive microenvironment by secreting sEVs’ HSP90, offering valuable insights into the involvement of HSP90 in exosome-mediated communication within the context of NF2-related schwannomatosis (NF2-SWN). This information has the potential to inform the design of effective immunotherapeutic protocols and offer new treatment options for NF2-SWN patients.

## 1. Introduction

NF2-related schwannomatosis (NF2-SWN) is an autosomal dominant disorder marked by the presence of bilateral vestibular schwannomas, along with other tumors and ocular abnormalities [1,2,3]. Despite the benign nature of these tumors, NF2-SWN patients experience substantial morbidity and mortality as a result of tumor positioning and the adverse effects of treatment [4,5]. Currently, the treatment options for these tumors are limited, underscoring the urgent need to explore innovative therapeutic alternatives.

Studies have revealed the significant influence of the tumor microenvironment on tumor progression, metastasis, and recurrence [6,7,8,9]. Our previous studies [10] have demonstrated that NF2-SWN patients exist in an immunosuppressive state, prominently marked by the involvement of myeloid-derived suppressor cells (MDSCs) in mediating immune suppression. Remarkably, our findings revealed that targeted neutralization of TGF-β with specific antibodies partially alleviated the inhibitory effects of MDSCs on T cells. These insights into the suppressive mechanisms initiated by NF2-associated tumors offer promising pathways for the development of effective immunotherapeutic strategies and potential therapeutic interventions for NF2-SWN patients.

Extracellular vehicles (EVs) serve as small cellular surrogates, transferring substantial amounts of material both locally and systemically [11,12,13]. SEVs have a significant impact on cancer. They facilitate the migration of cancer cells and contribute to the formation of new blood vessels. These small particles influence surrounding tissues and alter the functions of specific cells. Additionally, they assist tumors in evading the immune system by releasing molecules like PD-L1 and modifying cytokines to promote this process. This is crucial as it can lead to more aggressive cancer behavior and change the tumor’s response to treatment [14]. Accumulating studies have demonstrated that EVs play a pivotal role in regulating tumorigenesis and tumor progression by fostering an immunosuppressive microenvironment and enhancing tumor development through the transfer of vital cargoes, including nucleic acids, lipids, and proteins [15,16,17]. However, the microenvironment-modulating effects of NF2-EVs have yet to be explored.

In our current study, we purified sEVs from the culture medium of primary NF2-associated schwannoma cells and conducted a thorough proteomic analysis to investigate their potential roles in microenvironment modulation and tumor progression, as well as the underlying mechanisms involved. Understanding the impact of NF2-EVs on microenvironment dynamics is crucial for advancing immunotherapy and may pave the way for novel, effective treatments for NF2-SWN patients.

## 2. Materials and Methods

### 2.1. Patients

The study protocols received approval from Beijing Tiantan Hospital’s institutional review boards, and written consent was obtained from all participating patients. Patient samples were collected from individuals diagnosed with NF2-SWN, as confirmed by neuropathologists at Beijing Tiantan Hospital. For more details about the NF2-SWN patients, refer to Table 1.

### 2.2. Primary Schwannoma Cells Isolation and Culture

Primary cells were isolated using the methods described by Rosenbaum et al. [18]. Schwannoma tissues were dissected into smaller fragments and enzymatically digested with dispase grade II (1.25 U/mL, Roche, Basel, Switzerland) and collagenase type I (160 U/mL, Sigma, St. Louis, MO, USA). The resulting cells were suspended in DMEM supplemented with 10% FBS (Sigma), Pen/Strep (200 U/mL, Invitrogen, Carlsbad, CA, USA), forskolin (0.5μM, Tocris Bioscience, Bristol, UK), β1-heregulin (10 nM, R&D System, Minneapolis, MN, USA), and insulin (2.5 μg/mL, Sigma). These cells were then seeded into poly-L-lysine-coated (1 mg/mL, Sigma) and laminin-coated (4 μg/mL, Life Technologies, Carlsbad, CA, USA) 24-well plates. The medium was changed every four days, and the cells were passaged when reaching a confluence level of 70~80%.

### 2.3. SEVs Isolation

The cells were cultured in DMEM supplemented with 10% FBS until they reached 80% confluence. Once the desired confluence was achieved, the culture medium was carefully removed, and the cells were rinsed once with PBS. They were then incubated in serum-free medium for 48 h. Following this incubation period, the collected medium underwent a series of centrifugation steps. Initially, it was centrifuged at 300× *g* for 10 min at 4 °C, followed by a second centrifugation at 2000× *g* for 10 min at the same temperature. The supernatant obtained from this step was then subjected to further centrifugation at 10,000× *g* for 10 min at 4 °C. Finally, the resulting supernatant underwent centrifugation at an impressive 110,000× *g* for 2 h at 4 °C, yielding purified sEVs. These sEVs were subsequently resuspended in pre-cooled PBS.

### 2.4. Transmission Electron Microscopy

The morphology and approximate size of the isolated sEVs were assessed using Transmission Electron Microscopy (TEM, Gatan Inc., Pleasanton, CA, USA). The isolated sEVs were suspended in PBS and carefully applied to Formvar-coated copper grids. The prepared samples were then affixed to metal stubs and gold-coated to a thickness of 15 nm. The exosome sample was analyzed under a TEM microscope.

### 2.5. Nanoparticle Tracking Analysis (NTA)

The ZetaView (Particle Metrix, version 8.04.02) was utilized to perform a detailed absolute size distribution analysis of the sEVs. Illuminated by a laser, the sEVs scattered light, which was captured by a camera, creating a video file that showcased their Brownian motion. The ZetaView meticulously tracked and analyzed individual particles ranging from 10 to 1000 nm in size. Each sample underwent three recordings to ensure comprehensive analysis.

### 2.6. Analysis of MDSC Percentages in Co-Culture

The CD14^+^ monocytes were collected after being exposed for 24 h to NF2-EVs, and then filtered using 70 µm cell strainers (BD Falcon, BD Biosciences, San Jose, CA, USA). The resulting single-cell suspension was then re-suspended in FACS staining buffer, and stained with anti-HLA-DR Percp-cy5.5 (eBioscience, 0.015 µg/test, San Diego, CA, USA), anti-CD33 APC (eBioscience, 0.125 µg/test), and anti-CD11b FITC antibodies (eBioscience, 0.5 µg/test), along with corresponding isotype controls. Flow cytometry analysis was conducted using a (BD Biosciences, San Jose, CA, USA), and the data were processed with FlowJo software (version X10.0.7).

### 2.7. Detection of Intracellular ROS Generation

For the detection of intracellular ROS levels, ROS-sensitive probe DCFH-DA is used. Monocytes, both control and NF2 EV-treated, are resuspended in PBS, incubated with 5 µM of DCFH-DA in the dark for 30 min at 37 °C, and immediately analyzed with a flow cytometer.

### 2.8. Cell Viability Assay

Cell viability was evaluated using the Cell Counting Kit-8 (CCK8, Dojindo Laboratories, Kumamoto, Japan). In this process, cells were plated at a density of 1 × 10^4^ cells per well in 96-well plates and treated with either PBS or NF2-EVs for periods of 0, 24, or 48 h. At the conclusion of each treatment interval, 10 µL of CCK8 solution was added to each well and incubated for 2 h at 37 °C. The resulting color intensity was then measured at 450 nm using the SpectraMax M5 microplate reader (Molecular Devices, San Jose, CA, USA). Cell viability was expressed as a percentage relative to control cells cultured in NF2-exosome-free medium, which was designated as 100%. Each assay was conducted in triplicate to ensure reliability and accuracy.

### 2.9. Luminex Liquid Suspension Chip Detection

For the Luminex assay, the conditioned medium from stimulated MDSCs was collected. Wayen Biotechnologies facilitated the Luminex liquid suspension chip detection. We utilized the Bio-Plex Pro Human Cytokine Group I Panel 27-plex, following the manufacturer’s instructions meticulously to ensure optimal results.

### 2.10. Mass Spectrum Analysis

An Orbitrap Fusion Lumos mass spectrometer (Thermo Fisher Scientific Inc., Waltham, MA, USA), paired with the EASY-nLC 1000 liquid chromatography system (Thermo Fisher Scientific Inc.) was employed for a comprehensive analysis of proteins, encompassing both qualitative and quantitative dimensions. The dried samples were rehydrated using 0.1% formic acid (FA) and then separated on a custom-made analytical column measuring 75 μm I.D. × 25 cm, packed with C18 resin (C18, 1.9 μm, 120 Å, Maisch GmbH, Ammerbuch, Germany). The flow rate was meticulously set to 300 nL/min. Full scans were conducted over a mass-to-charge ratio (m/z) range of 350 to 1800, achieving a remarkable mass resolution of 60,000 (FWHM). Precursor ions were fragmented through high-energy collision dissociation (HCD) at a normalized collision energy (NCE) of 30%, ensuring precise and detailed analysis.

### 2.11. Western Blot

Total proteins from different treated cells were extracted using Radio immunoprecipitation assay lysis buffer. After measuring the protein concentration, 20–40 μg of protein per sample were separated by 10% SDS-polyacrylamide gel electrophoresis (SDS-PAGE) and then transferred to polyvinylidene difluoride membrane. The membranes were then blocked at room temperature for 2 h with 5% non-fat milk and incubated at 4 °C overnight with diluted (1:1000) primary antibodies against CD9 (Proteintech, 60232-1-Ig, Wuhan, China), CD81 (Proteintech, 66866-1-Ig), TOMM20 (Proteintech, 11802-1-AP), HSP90 (Proteintech, 13171-1-AP), AKT (CST, #9272S, Danvers, MA, USA), p-AKT (CST, #4051S), ERK (CST, #4695), p-ERK (CST, #9101S), following by incubation with appropriate HRP-conjugated secondary antibody and detection by Western-Light Chemiluminescent Detection System. The Image J software (version 1.51) was used to analyze the relative density of immunoreactive bands. The densitometry data for all Western results can be found in Table S1.

### 2.12. PPI Regulatory Network Analysis

We used the STRING database (version 11.0) (https://cn.string-db.org/) to construct a PPI network to show the relationships of the DeMiRNAs. A comprehensive score over 0.9 was used to indicate statistical significance, and we hid nodes that were not connected with other entities in the network. Cytoscape (version 3.7.2) was used to construct the network, and the hub genes in the PPI network were analyzed using cytohubba in Cytoscape (version 3.7.2).

### 2.13. Statistical Analysis

Differences in quantitative normally distributed variables between 2 groups were tested with Student’s *t*-test and *p* values less than 0.05 were considered to indicate statistical significance. Data are expressed as the Mean ± SD, and analyses were performed using GraphPad Prism (version 9.5.0).

## 3. Results

### 3.1. Patient Characteristics

The clinical information of NF2-SWN patients is summarized in Table 1. Among the 11 NF2-SWN patients, 5 (45.45%) are male and 6 (54.55%) are female. The age at the time of diagnosis ranged from 14 and 46 years, with a mean age of 28 years (standard deviation [SD] ± 14.02 years).

### 3.2. Characterization of NF2-EVs

**Figure 1:**
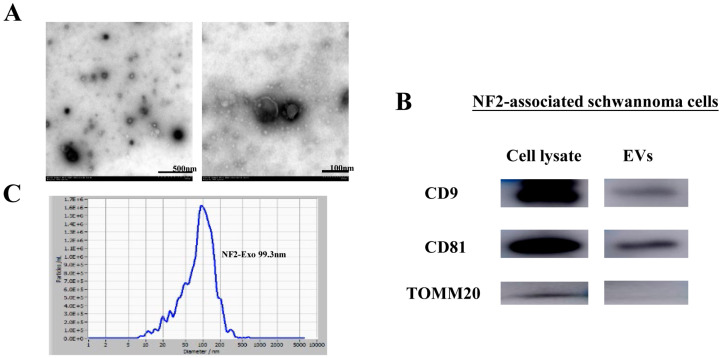
NF2-associated schwannoma cells secrete sEVs. (**A**). NF2-EVs showed typical double-layered membrane structure observed under the electron microscope. (**B**). NF2-EVs expressed sEVs’ labeled proteins CD9 and CD81, and control marker TOMM20. (**C**). NF2-EVs ranged in size from 50 to 200 nm.

SEVs were isolated from the supernatant of primary cell cultures derived from NF2-associated schwannoma tissues. The purity of primary cells was identified through SOX100 staining, and the results are attached in Figure S1. Transmission electron microscopy unveiled vesicles exhibiting the characteristic double-layered membrane structure of sEVs (Figure 1A). Protein analysis via western blotting confirmed the presence of CD81 and CD9 in NF2-EVs, and the mitochondrial marker TOMM20 as control (Figure 1B). The whole blots (uncropped blots) showing all the bands with all molecular weight markers have been included in the Supplementary Materials. The size distribution analysis of the sEVs was performed using ZetaView (version 8.04.02), indicating that sEVs derived from NF2-associated schwannoma ranged in size from 50 to 200 nm (Figure 1C).

### 3.3. Monocytes Co-Cultured with NF2-EVs Resemble MDSC Cells in Phenotype and Function

We previously identified HLA-DR^−^CD33^+^CD11b^+^ cells as a potential population of MDSCs in NF2-SWN patients, which exert immunosuppressive effects on T cells. Furthermore, we discovered that tumor cell supernatants could induce the transformation of monocytes into an MDSC phenotype. Recognizing the crucial role of tumor-derived EVs in these supernatants, we aimed to investigate whether tumor cells promote the transformation of monocytes into MDSCs through the secretion of EVs.

**Figure 2:**
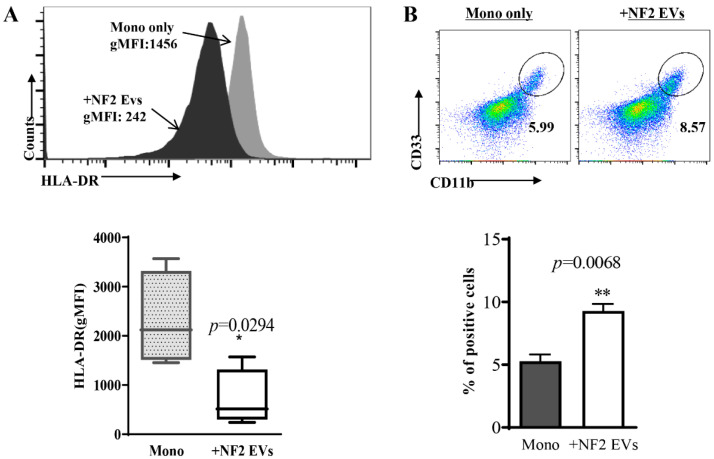
Monocytes (Mono) co-cultured with NF2-EVs resemble MDSC cells in phenotype. (**A**). Cytometric (up) and statistical (down) analysis of HLA-DR expression in CD14^+^ monocytes after exposure for 24 h to NF2-EVs. (**B**). NF2-EVs induced CD14^+^ monocytes transformation into an HLA-DR^−^CD33^+^CD11b^+^ cell phenotype. * *p* < 0.05, ** *p* < 0.01 compared to the Mono group.

**Figure 3:**
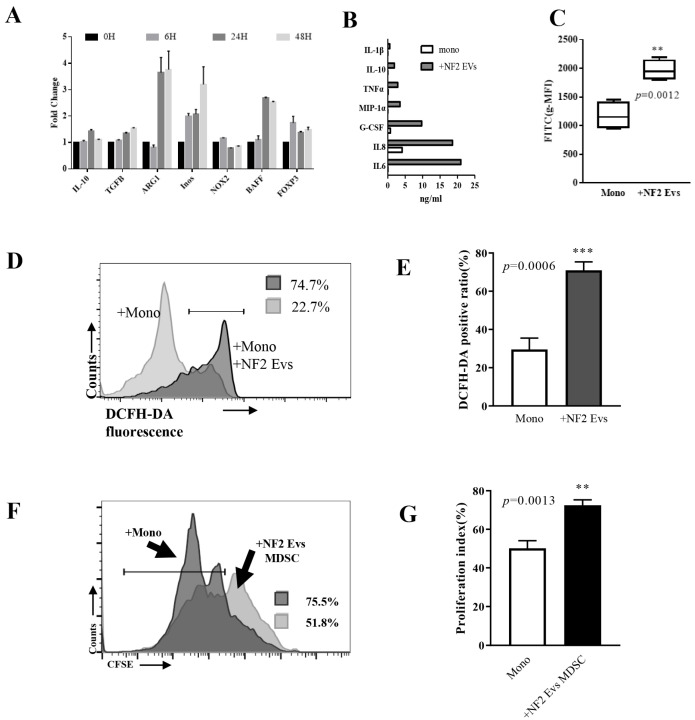
Monocytes (Mono) co-cultured with NF2-EVs resemble MDSC cells in function. (**A**). HLA-DR^−^CD33^+^CD11b^+^ cells induced by NF2-EVs (NF2-EVs-MDSCs) show increase in *ARG1*, *iNOS*, and *BAFF* transcription. (**B**). NF2-EVs-MDSCs show increase in IL-10, TNFα, MIP-1α, G-CSF, IL-8, and IL-6 secretion. (**C**–**E**). NF2-EVs-MDSCs can produce a larger amount of reactive oxygen species (ROS). (**F**,**G**). NF2-EVs-MDSCs were also able to inhibit autologous T cell proliferation. ** *p* < 0.01, *** *p* < 0.005 compared to the Mono group.

In this study, we found that CD14^+^ monocytes exposed to NF2-EVs for 24 h displayed a marked downregulation of HLA-DR expression (Figure 2A) and an increased percentage of HLA-DR^−^CD33^+^CD11b^+^ cells (Figure 2B). The HLA-DR^−^CD33^+^CD11b^+^ cells induced by NF2-EVs (termed NF2-EVs-MDSCs) exhibited elevated transcription levels of *ARG1*, *iNOS*, and *BAFF* (Figure 3A), along with increased secretion of IL-10, TNFα, MIP-1α, G-CSF, IL-8, and IL-6 (Figure 3B). Additionally, these cells produced a significantly greater amount of reactive oxygen species (ROS) (Figure 3C–E). Notably, the presence of NF2-EVs-MDSCs inhibited the proliferation of autologous T cells (Figure 3F,G). Collectively, these findings demonstrate that NF2-EVs can induce the transformation of monocytes into MDSCs, highlighting their role as key players in the immunosuppressive microenvironment associated with NF2-SWN.

### 3.4. NF2-EVs Enhance NF2-Associated Schwannoma Cells Proliferation

**Figure 4:**
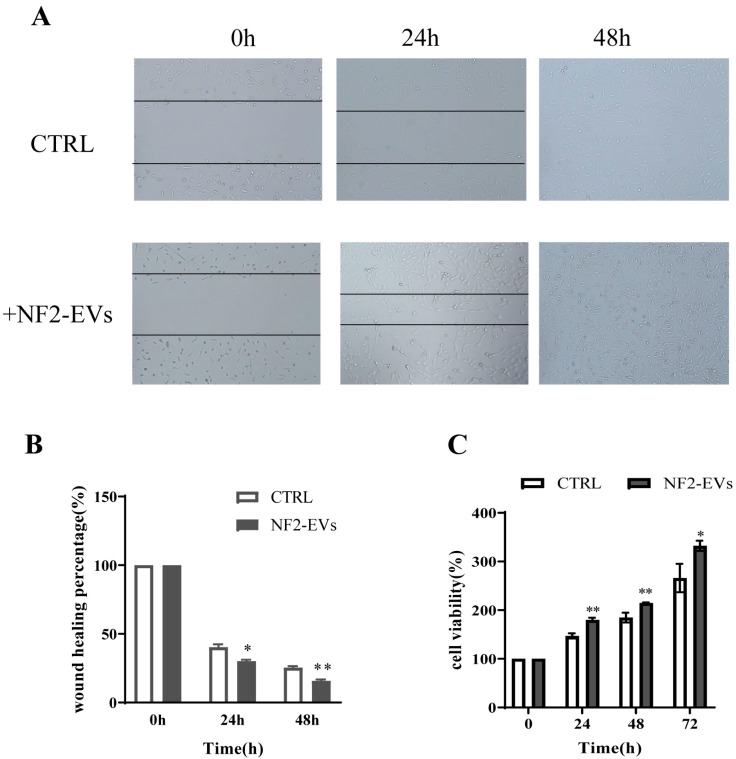
NF2-EVs enhance NF2-associated schwannoma cells’ proliferation. (**A**,**B**). NF2-EVs promoted NF2-associated schwannoma cell wound closing (**C**). NF2-EVs obviously promoted the proliferation of NF2-associated schwannoma cells in vitro. * *p* < 0.05, ** *p* < 0.01 compared to the CTRL group.

Next, we evaluated the impact of NF2-EVs on the proliferation of NF2-associated schwannoma cells. Our findings revealed that NF2-EVs not only facilitated the wound healing process in these cells (Figure 4A,B) but also significantly enhanced their proliferation in vitro compared to the control group without NF2-EVs (Figure 4C). These results underscore the potent role of NF2-EVs in promoting the proliferation of NF2-associated schwannoma cells.

### 3.5. Exosomal HSP90 Is a Key Modulator in NF2-Associated Tumor Progression and Immunity

**Figure 5:**
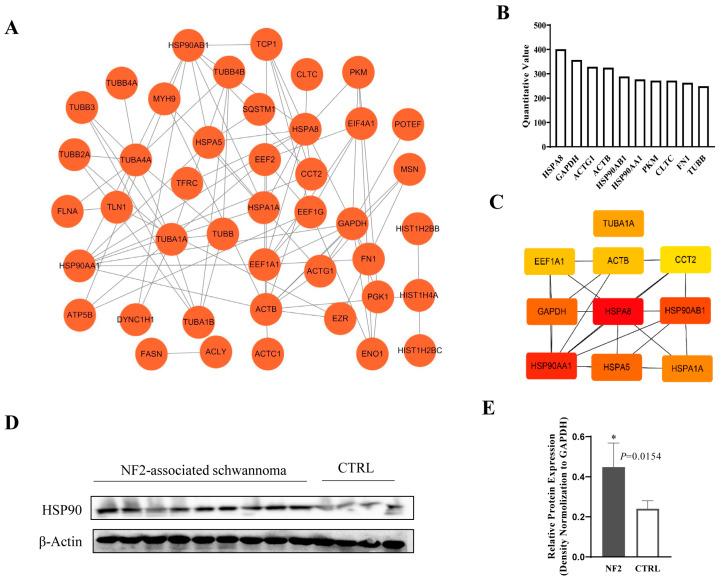
Identification of HSP90 in NF2-EVs. (**A**). The network of differentially expressed proteins obtained by STRING analysis. (**B**). The top10 most abundantly expressed proteins in the NF2-EVs. (**C**). The top-10 key hub nodes ranked by MCC method. (**D**). Western blot analysis of HSP90 in NF2-associated schwannoma tissues and normal peripheral nerve control. F. Statistical analysis of HSP90 expression. * *p* < 0.05 compared to the CTRL group.

To pinpoint the specific components within NF2-EVs that enhance MDSC transformation and stimulate the proliferation of NF2-associated schwannoma cells, we utilized mass spectrometry screening on NF2-EVs. Utilizing the STRING online database (version 11.3) alongside Cytoscape software (version 3.7.2), we meticulously analyzed and visualized the protein-protein interaction (PPI) network, setting a threshold score of >0.9 (Figure 5A). Our results revealed that the ten most abundantly expressed proteins in NF2-EVs include HSPA8, GAPDH, ACTG1, ACTB, HSP90AB1, HSP90AA1, PKM, CLTC, FN1, and TUBB (Figure 5B). Among these, the top ten key hub nodes—ranked by the Maximum Clique Centrality (MCC) method—were HSPA8, HSP90AA1, HSP90AB1, GAPDH, HSPA5, HSPA1A, TUBA1A, EEF1A1, ACTB, and CCT2, as illustrated in Figure 5C.

After conducting a literature review, we narrowed our focus to two most abundantly expressed proteins, HSPA8 and HSP90. Further Western blot analysis of HSP90 in NF2-associated schwannoma tissues provided further confirmation that HSP90 was significantly highly expressed (Figure 5D,E). The whole blots (uncropped blots) showing all the bands with all molecular weight markers have been included in the Supplementary Materials. Based on our findings, we hypothesized that HSP90 could be one of the key functional factors within NF2-EVs. Consequently, we selected HSP90 for further analysis and investigation.

**Figure 6:**
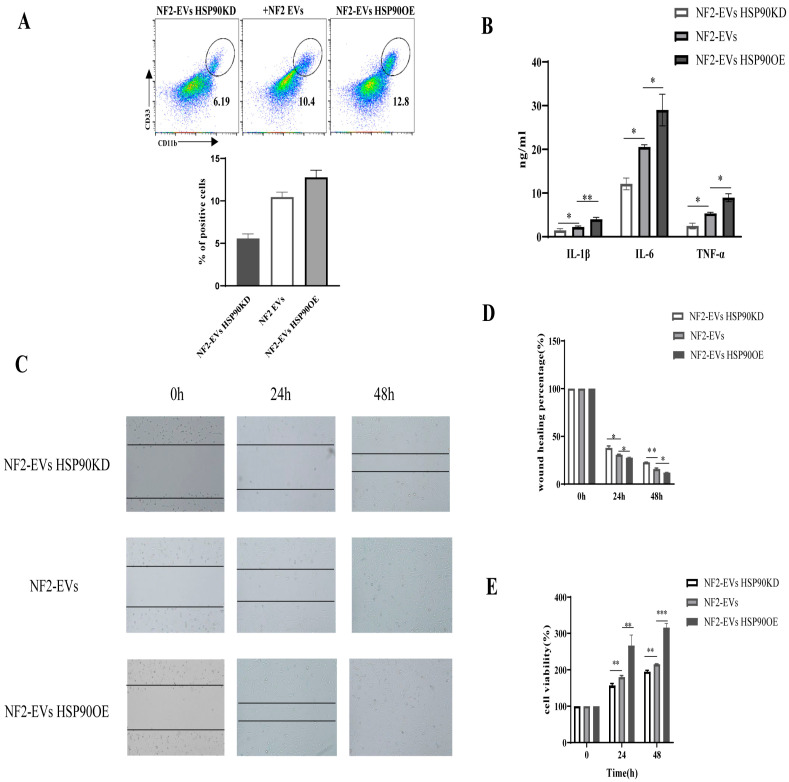
Exosomal HSP90 is a key modulator in NF2-associated tumor progression and immunity. (**A**): Cytometric analysis of HLA-DR^−^CD33^+^CD11b^+^ cells percentage in monocytes when coculture with NF2-EVs ^HSP90KD^ or NF2-EVs ^HSP90OE^. (**B**): Cytokine secretion in the supernatants of monocytes when cocultured with NF2-EVs ^HSP90KD^ or NF2-EVs ^HSP90OE^. (**C**,**D**). NF2-EVs ^HSP90OE^ promoted NF2-associated schwannoma cell wound closing. (**E**). NF2-EVs ^HSP90OE^ obviously promoted the proliferation of NF2-associated schwannoma cells in vitro. * *p* < 0.05, ** *p* < 0.01, *** *p* < 0.005 compared to the NF2-EVs HSP90KD group.

We conducted experiments to knock down and overexpress HSP90 in NF2-associated schwannoma cells, subsequently extracting sEVs (designated as NF2-EVs ^HSP90KD^ and NF2-EVs^HSP90OE^) from their supernatants. We then repeated the co-culture experiments to assess the effects of these modifications. The results revealed that following HSP90 downregulation, the percentage of HLA-DR^−^CD33^+^CD11b^+^ cells significantly decreased when monocytes were co-cultured with NF2-EVs ^HSP90KD^, compared to the NF2-EVs group. Conversely, HSP90 overexpression markedly increased the percentage of HLA-DR^−^CD33^+^CD11b^+^ cells during co-culture with NF2-EVs^HSP90OE^ (Figure 6A). Additionally, the secretion of IL-1β, IL-6, and TNF-α in the co-culture supernatants was significantly diminished in the presence of NF2-EVs ^HSP90KD^, while these cytokine levels were enhanced when monocytes were co-cultured with NF2-EVs HSP90OE (Figure 6B). These findings underscore the crucial role of HSP90 in the transformation of MDSCs.

Building on these results, we further investigated the impact of sEV’s HSP90 on the proliferation of NF2-associated schwannoma cells. Our study demonstrated that the proliferation ability was significantly higher in the NF2-EVs ^HSP90OE^ group compared to the other groups (NF2-EVs and NF2-EVs ^HSP90KD^) (Figure 6C–E). These findings provide compelling evidence for the functional role of sEV’s HSP90 in promoting the proliferation of NF2-associated schwannoma cells.

### 3.6. HSP90 Modulates NF2-Associated Tumor Progression and Immunity by Regulating AKT

**Figure 7:**
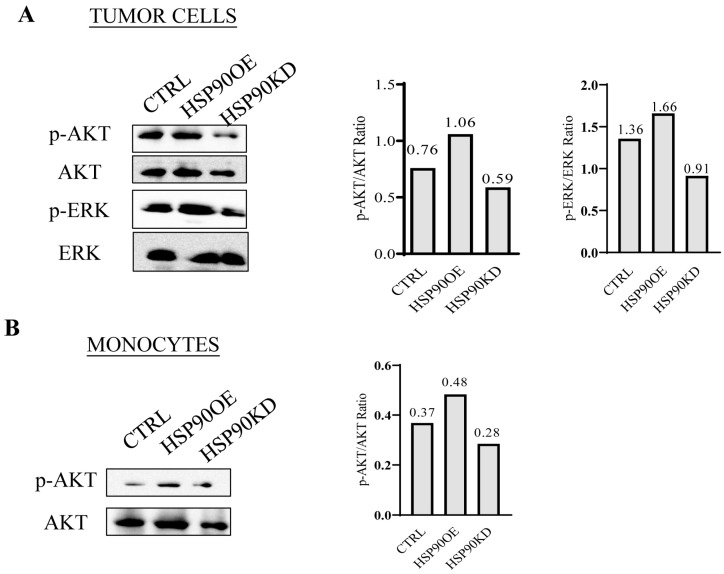
HSP90 modulates NF2-associated tumor progression and immunity by regulating AKT. (**A**): **Left**: Western blot analysis of pAKT and pERK expression in NF2-associated schwannoma cells after HSP90 knockdown or overexpression. **Right**: Bar graph of the densitometric ratios for the corresponding bands. (**B**): **Left**: Western blot analysis of pAKT expression in monocytes after HSP90 knockdown or overexpression. **Right**: Bar graph of the densitometric ratios for the corresponding bands.

Next, we sought to explore the mechanism by which sEV’s HSP90 facilitates the proliferation of NF2-associated tumor cells and drives the transformation of MDSCs. To achieve this, we focused on NF2-related pathway genes and assessed their expression levels in NF2-associated schwannoma cells with either HSP90 knocked down or overexpressed. Our analysis revealed a correlation between HSP90 expression and the activation of pAKT and pERK. Notably, treatment with NF2-EVs ^HSP90OE^ significantly enhanced AKT phosphorylation in monocytes. Conversely, when exposed to NF2-EVs ^HSP90OE^, we observed a marked decrease in AKT phosphorylation (Figure 7). The whole blots (uncropped blots) showing all the bands with all molecular weight markers have been included in the Supplementary Materials. These findings underscore the pivotal role of sEV’s HSP90 as a key modulator in the progression of NF2-associated tumors and immune responses, primarily by activating AKT phosphorylation.

## 4. Discussion

In this study, we uncovered that NF2-EVs exhibit the characteristic morphology of sEVs, express specific sEV markers such as CD81 and CD9, with a size distribution ranging from 50 to 200 nm. Notably, we discovered that NF2-EVs can induce the phenotypic transformation of CD14^+^ monocytes into MDSC cells, thereby exerting potent immunosuppressive functions. Additionally, these vesicles significantly enhance the proliferative capacity of NF2-associated schwannoma cells. Through proteomic analysis and subsequent identification, we revealed that sEV’s HSP90 plays a crucial role in shaping the proliferation and immunosuppressive environment of NF2-associated tumors by modulating the AKT phosphorylation. In summary, our findings suggest that NF2-EVs can intricately influence tumor progression and immune responses in NF2-associated tumors through the transfer of HSP90.

SEVs are extracellular vesicles measuring 30–150 nm that are released by almost all cell types, including cancer cells. These sEVs, originating from cancer cells, contain a variety of molecular components such as proteins, mRNAs, and microRNAs. When transferred to recipient cells, they play a role in cancer progression, angiogenesis, metastasis, and immune evasion. SEVs play a pivotal role in shaping the tumor microenvironment, with their contents closely intertwined with cancer development [19,20]. Our study reveals that NF2-EVs not only facilitate the transformation of monocytes into MDSC-like cells but also amplify the proliferation of tumor cells, highlighting their critical importance in the progression of NF2-associated tumors.

To gain deeper insights into the biological properties and activities of NF2-EVs within the tumor microenvironment, we conducted a comprehensive proteomic analysis of these tumor-derived extracellular vesicles using mass spectrometry. Our findings identified HSP90 as one of the top ten key hub nodes within the protein expression profile of NF2-EVs. Subsequent Western blot analysis further validated that HSP90 is markedly upregulated in NF2-associated schwannoma tissues.

Through a series of gain and loss of function experiments, we uncovered that HSP90 plays a vital role in both the transformation of MDSCs and the proliferation of NF2-associated schwannoma cells. This discovery positions sEV’s HSP90 as a crucial modulator in the progression and immune landscape of NF2-associated tumors.

HSP90, a ubiquitous molecular chaperone, plays an essential role in maintaining specific proteins that are pivotal to cell proliferation and transformation [21,22]. Inhibition of HSP90 has been shown to trigger the degradation of its client proteins via the proteasomal pathway, making it an enticing therapeutic strategy that can simultaneously target multiple signaling pathways [23,24,25,26]. Previous studies have demonstrated significant antitumor efficacy of HSP90 inhibition in NF2-deficient cells, both in in *vitro* studies and animal models [27]. Furthermore, HSP90 inhibition has been found to effectively prevent the generation of MDSCs induced by melanoma cells [28,29], underscoring its critical role in the transformation of MDSCs.

Our study revealed that HSP90 is highly expressed in NF2-EVs and plays a pivotal role in enhancing the proliferation of NF2-associated schwannoma cells while also promoting the transformation of MDSCs in NF2-SWN. Further analysis demonstrated that HSP90 modulates the progression and immunity of NF2-associated tumors by regulating the AKT signaling pathway.

In summary, our findings highlight the important role of sEV’s HSP90 in regulating both the immune response and the proliferation of NF2-associated schwannoma cells. Released by these cells, sEV’s HSP90 can influence immunity and cellular growth through the modulation of the AKT phosphorylation. Our study illuminates a novel function of sEV’s HSP90 in regulating the progression of NF2-SWN, providing valuable insights into its involvement in exosome-mediated communication within this context. This knowledge holds significant potential to guide the development of effective immunotherapeutic strategies and pave the way for new treatment options for individuals affected by NF2-SWN.

## Figures and Tables

**Table 1:** Details of NF2-SWN patients.

Details of NF2-SWN Patients	Numbers (%)
All patients (*n* = 11)	
Median age (±SD)	28 ± 14.02
Gender	
Male	5 (45.45%)
Female	6 (54.55%)

## Data Availability

The original contributions presented in this study are included in the article/Supplementary Materials. Further inquiries can be directed to the corresponding author(s).

## Supplementary Materials

Supplementary Materials: The following supporting information can be downloaded at: https://www.mdpi.com/1718-7729/32/10/569/s1, File S1: Uncropped blots images for Figure 1, Figure 5 and Figure 7; Table S1: Densitometry; Figure S1: Purity assessment of primary cells.
