# Supplementary material for: Small Extracellular Vesicles Derived from NF2-Associated Schwannoma Cells Modulate Tumor Progression and Immunity via HSP90
Source: Curr Oncol. 2025 Oct 13;32(10):569. doi: 10.3390/curroncol32100569 (PMC12563180; doi:10.3390/curroncol32100569)

Fig1B-CD9

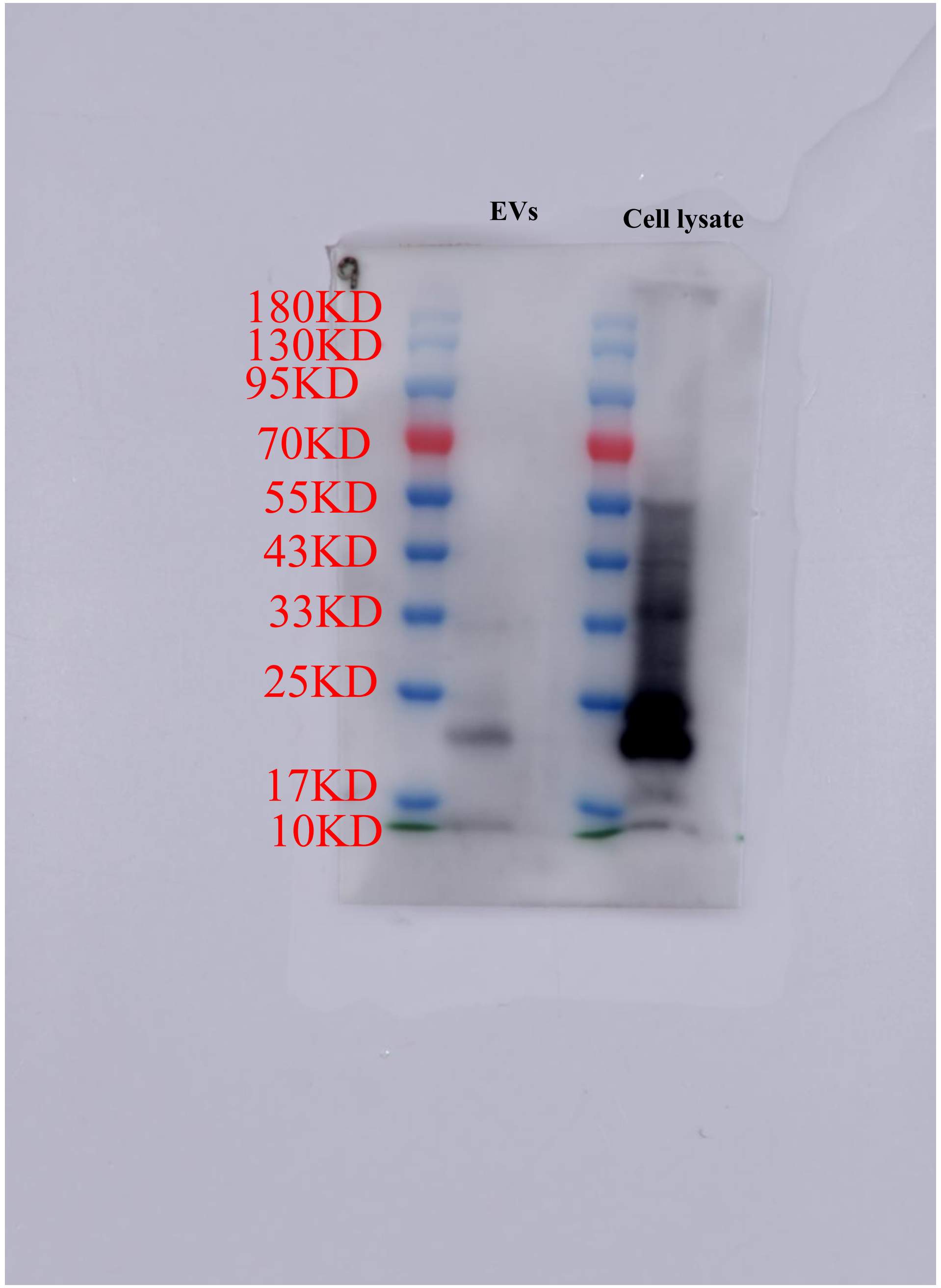

EVs
Cell lysate
180KD
130KD
95KD
70KD
55KD
43KD
33KD
25KD
17KD
10KD

Fig1B-CD81

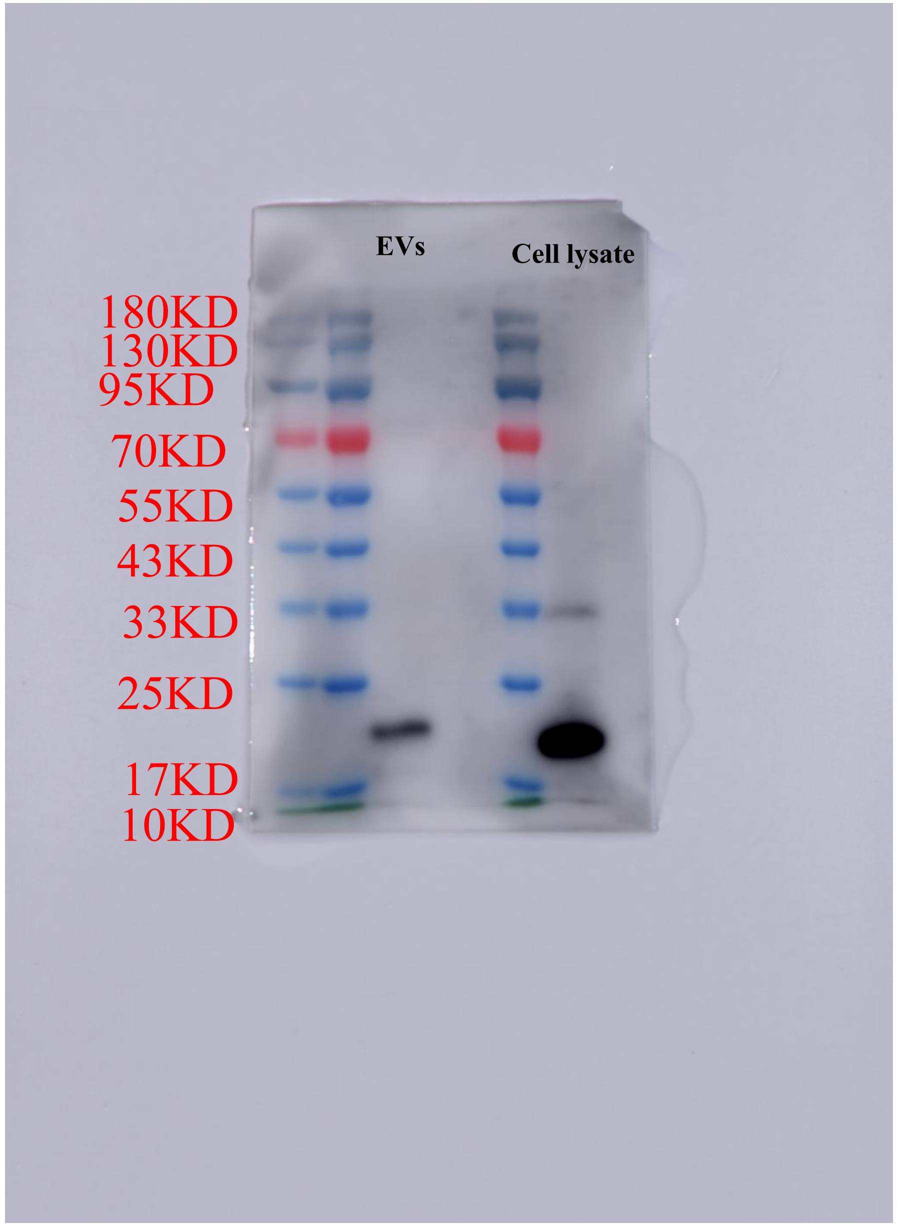
EVs
Cell lysate
180KD
130KD
95KD
70KD
55KD
43KD
33KD
25KD
17KD
10KD

Fig1B-TOMM20

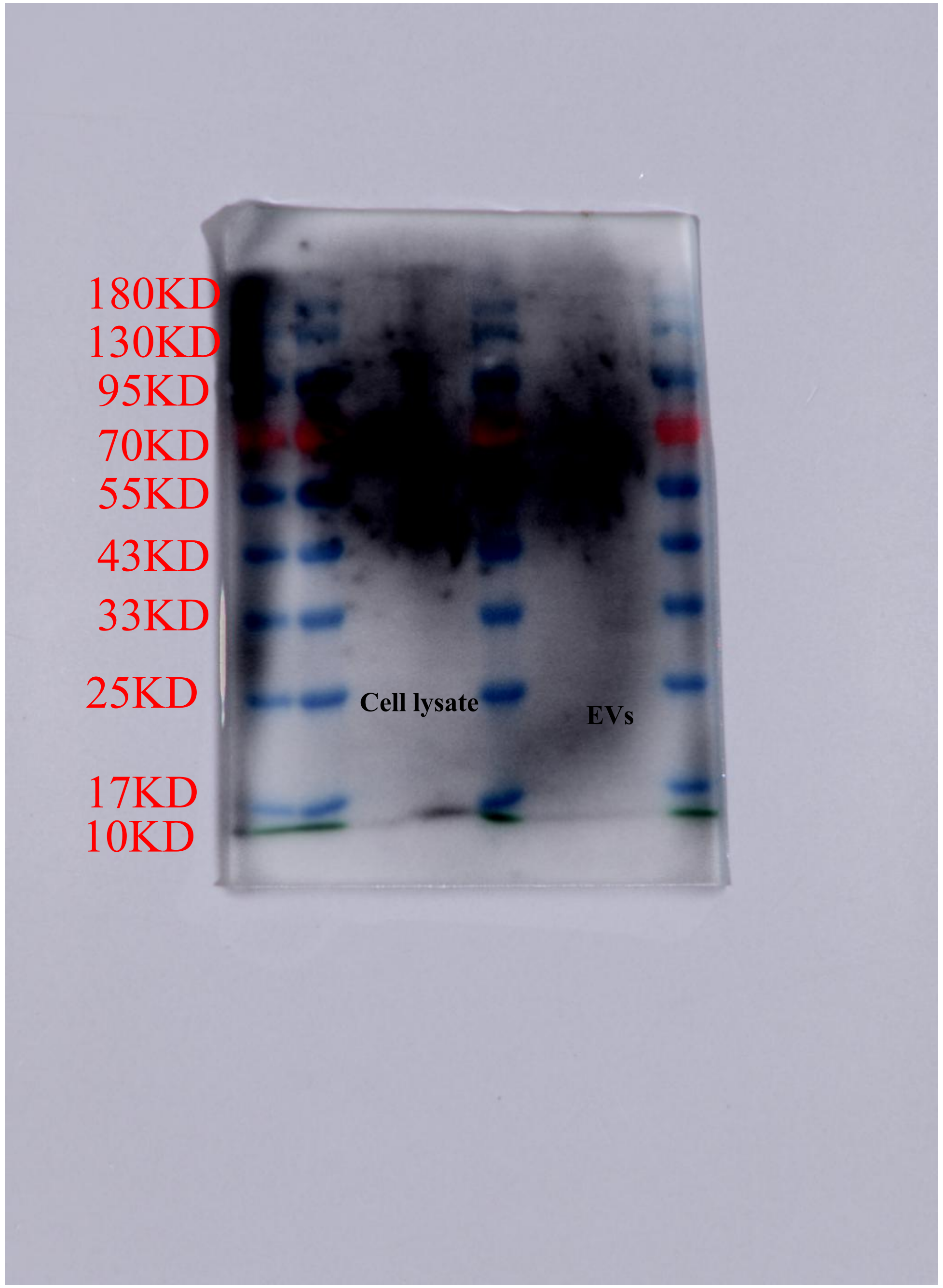

Fig5D-gapdh

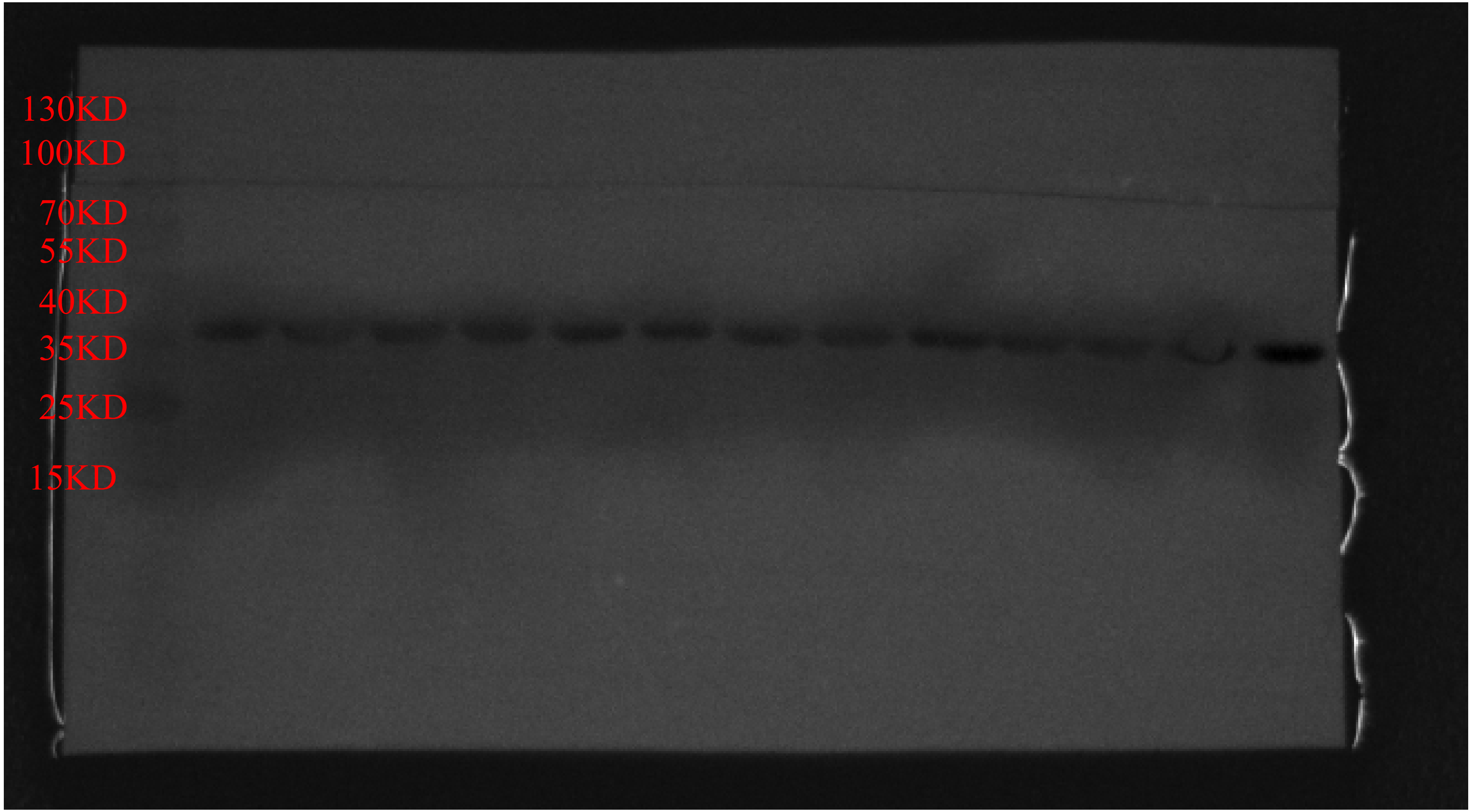

Fig5D-HSP90

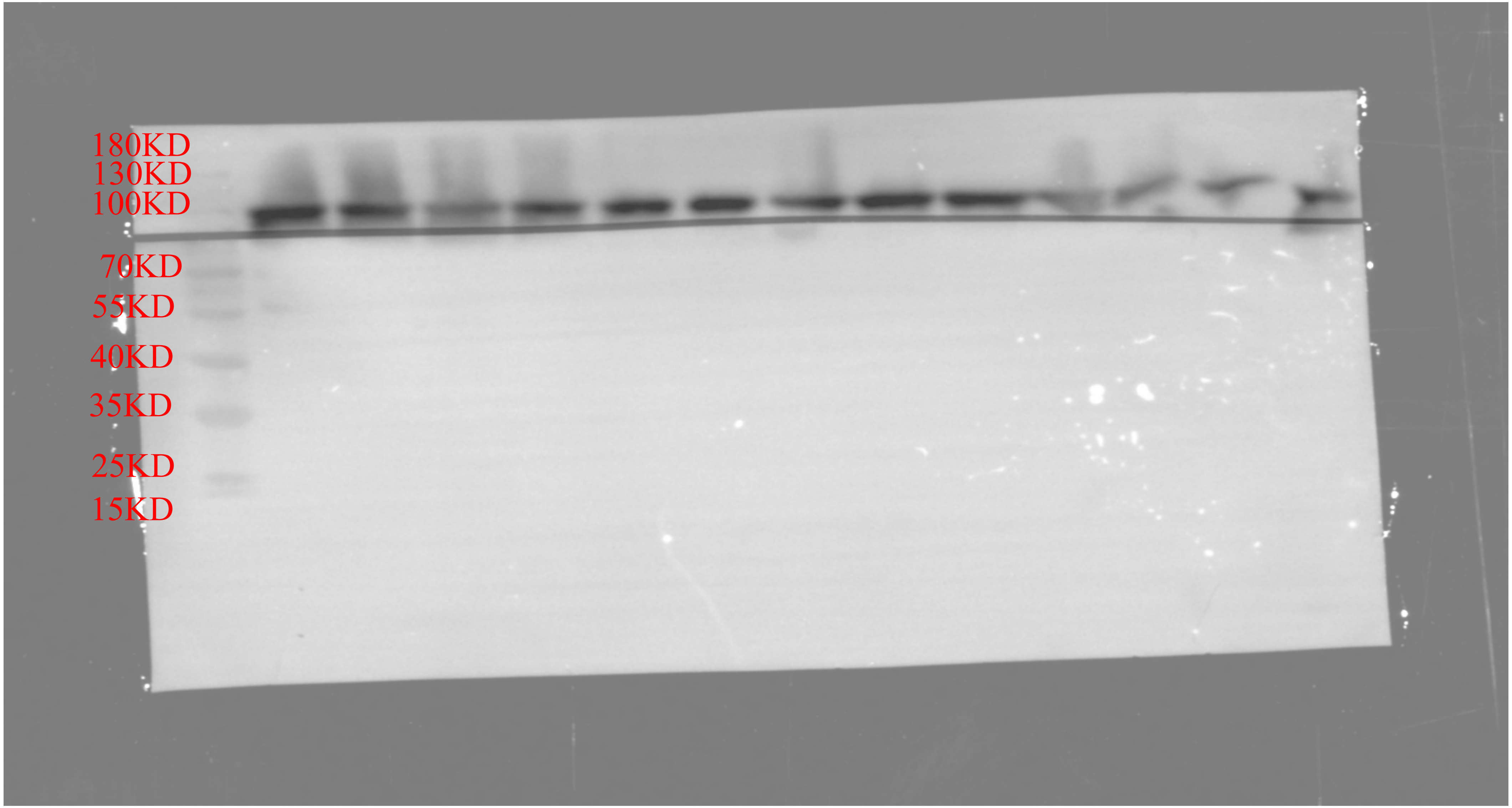

Fig7A-AKT

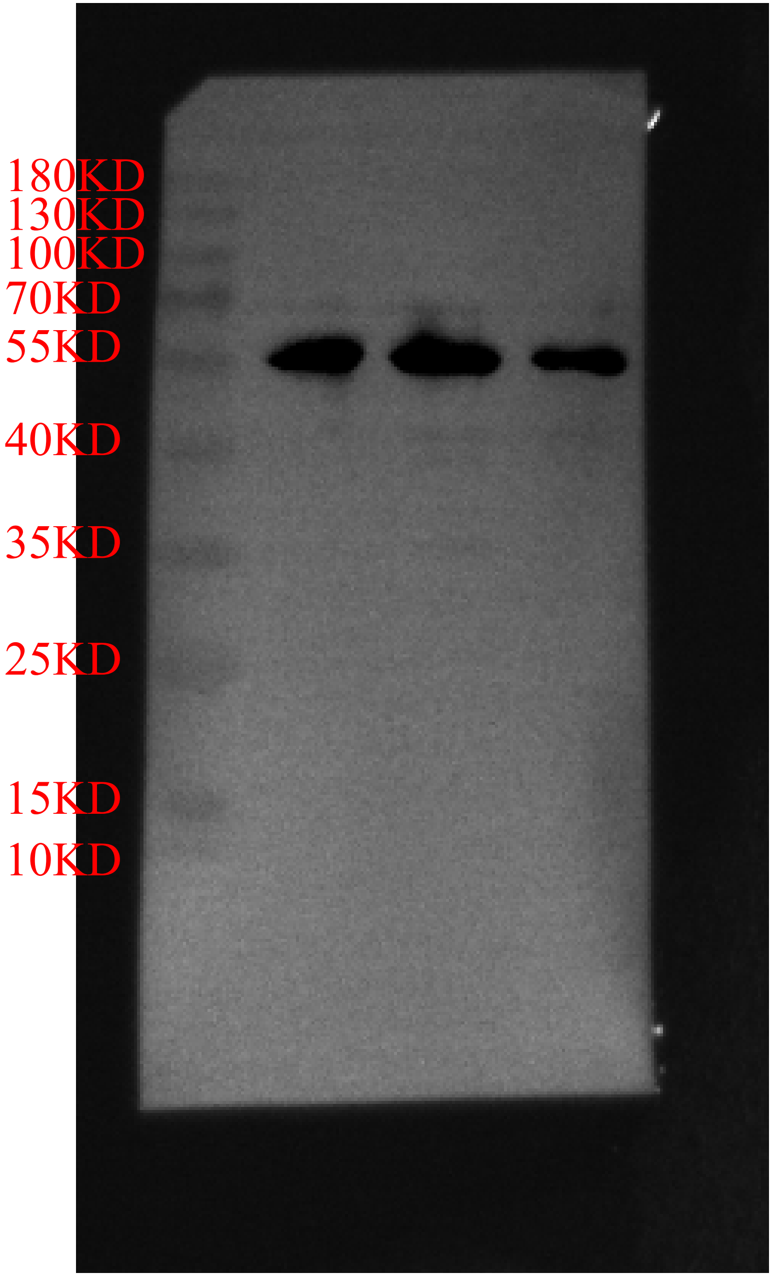

Fig7A-ERK

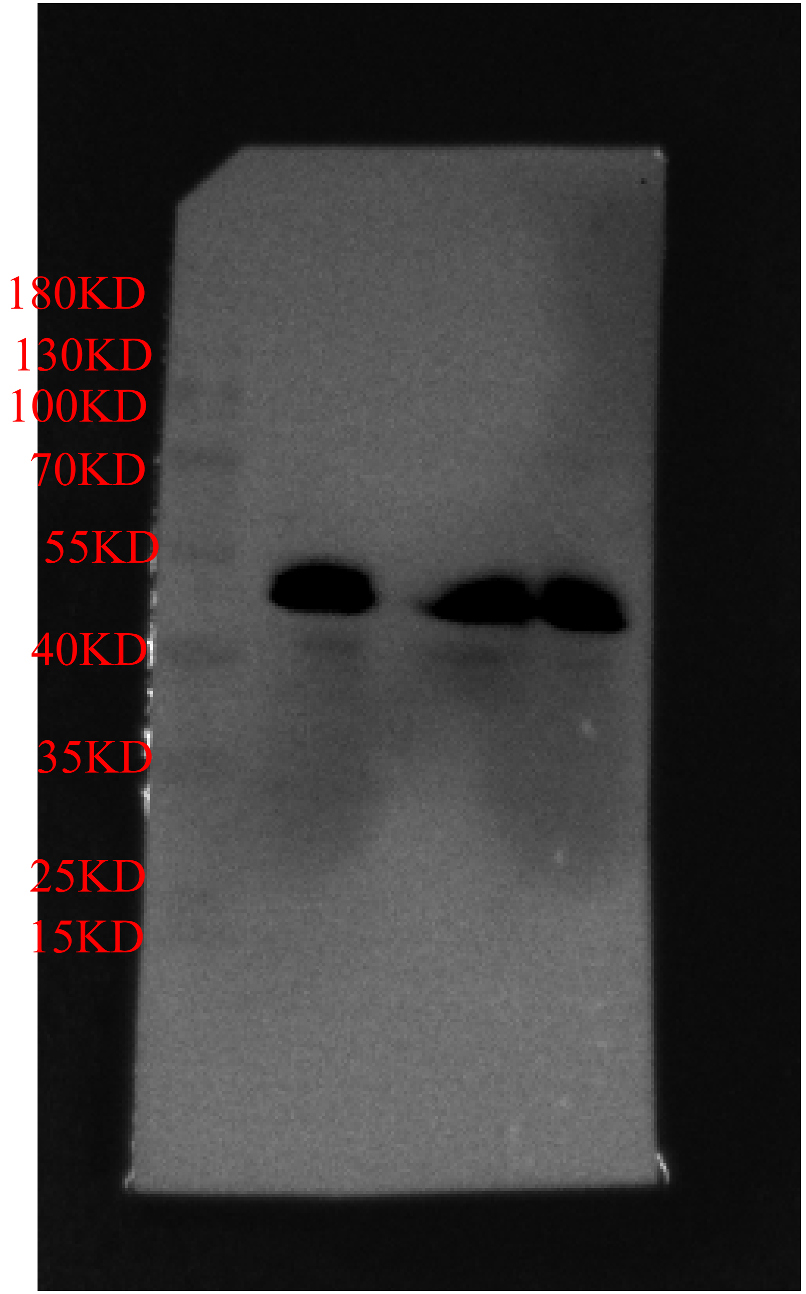

Fig7A-p-AKT

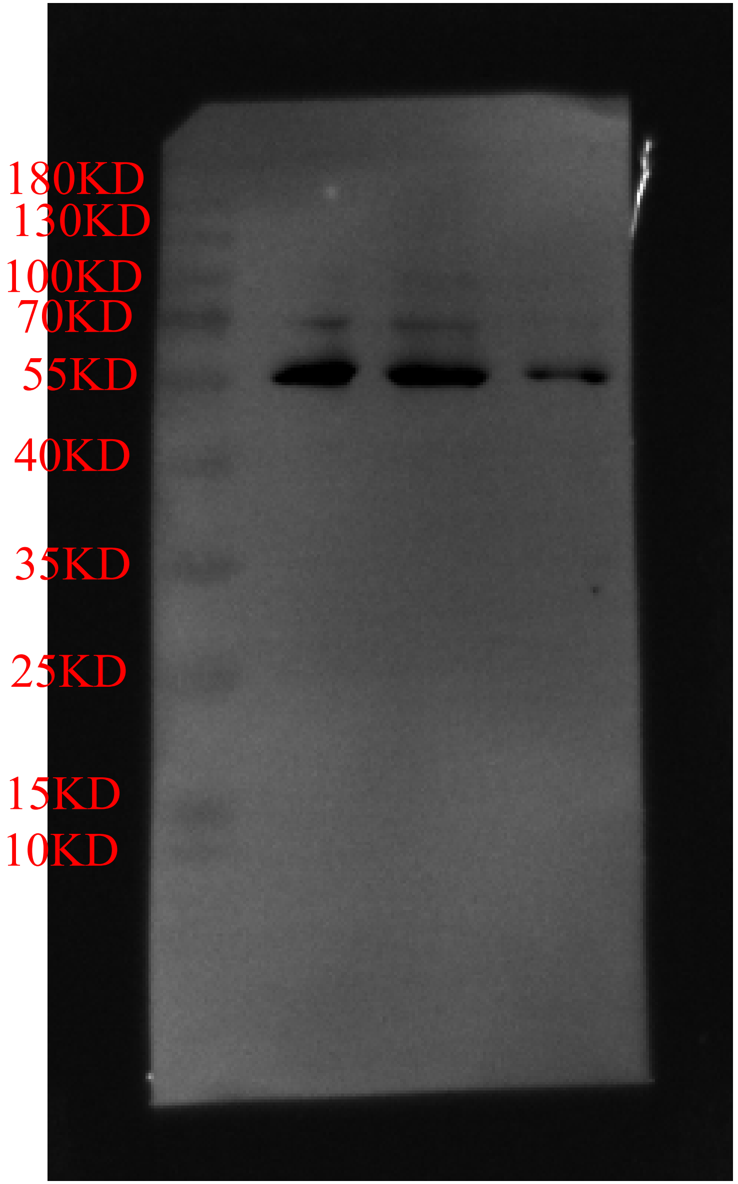

Fig7A-p-ERK

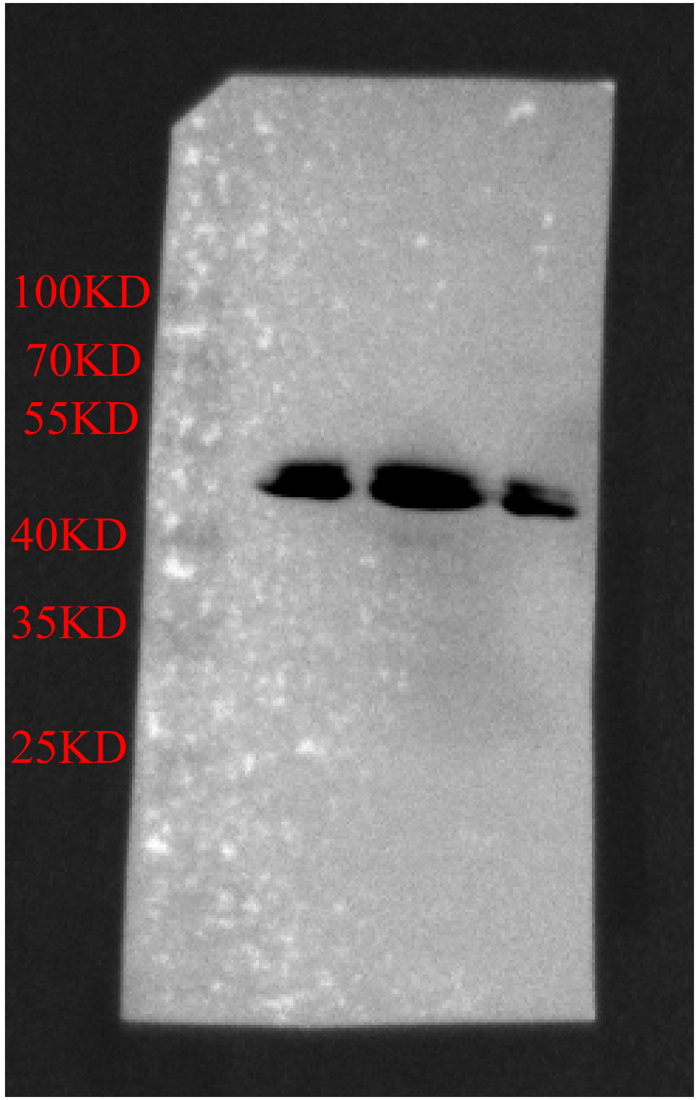

Fig7B-AKT

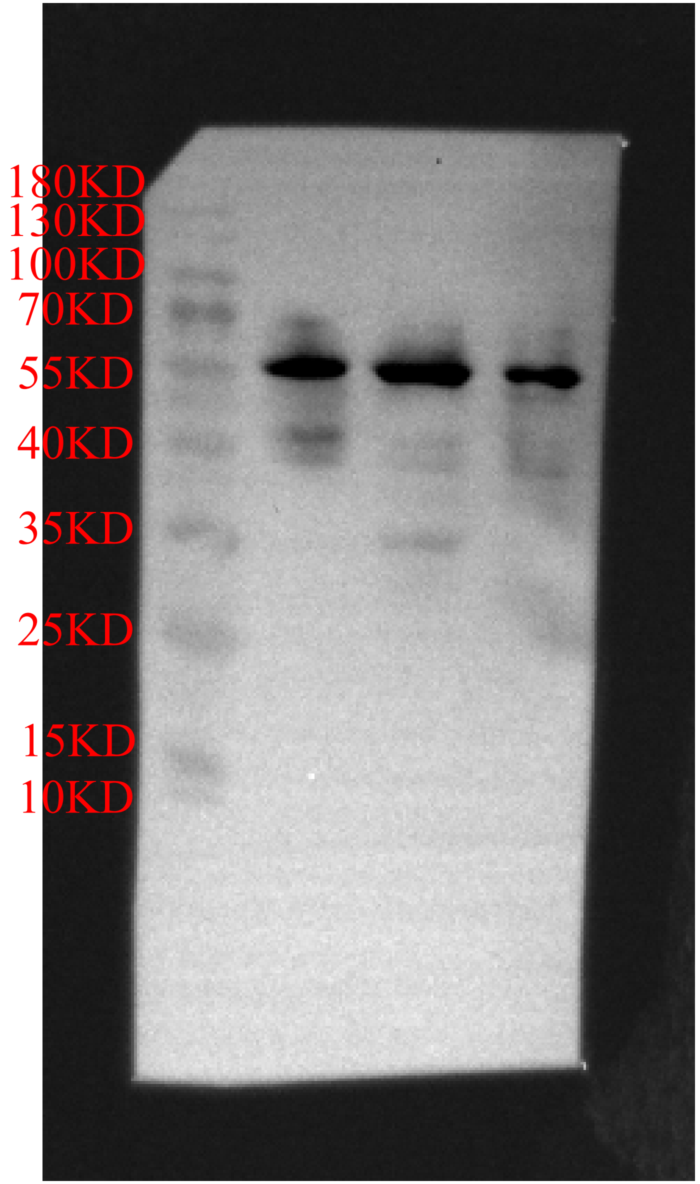

Fig7B-p-AKT

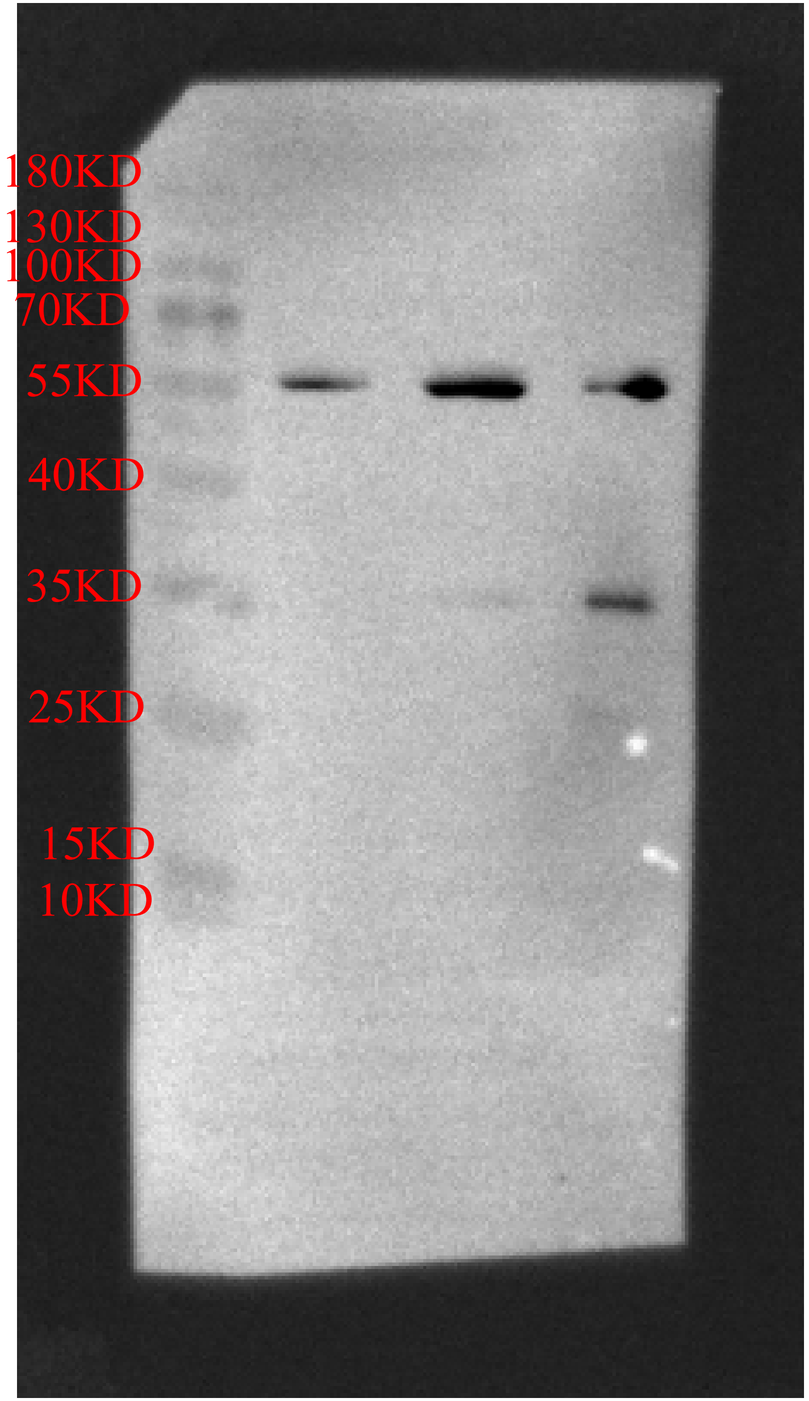

Supplement: Supplementary file 1 [file curroncol-32-00569-s001.zip › supplemental materials.pdf]
