# Supplementary figures and images for: Small Extracellular Vesicles Derived from NF2-Associated Schwannoma Cells Modulate Tumor Progression and Immunity via HSP90
Source: Curr Oncol. 2025 Oct 13;32(10):569. doi: 10.3390/curroncol32100569 (PMC12563180; doi:10.3390/curroncol32100569)

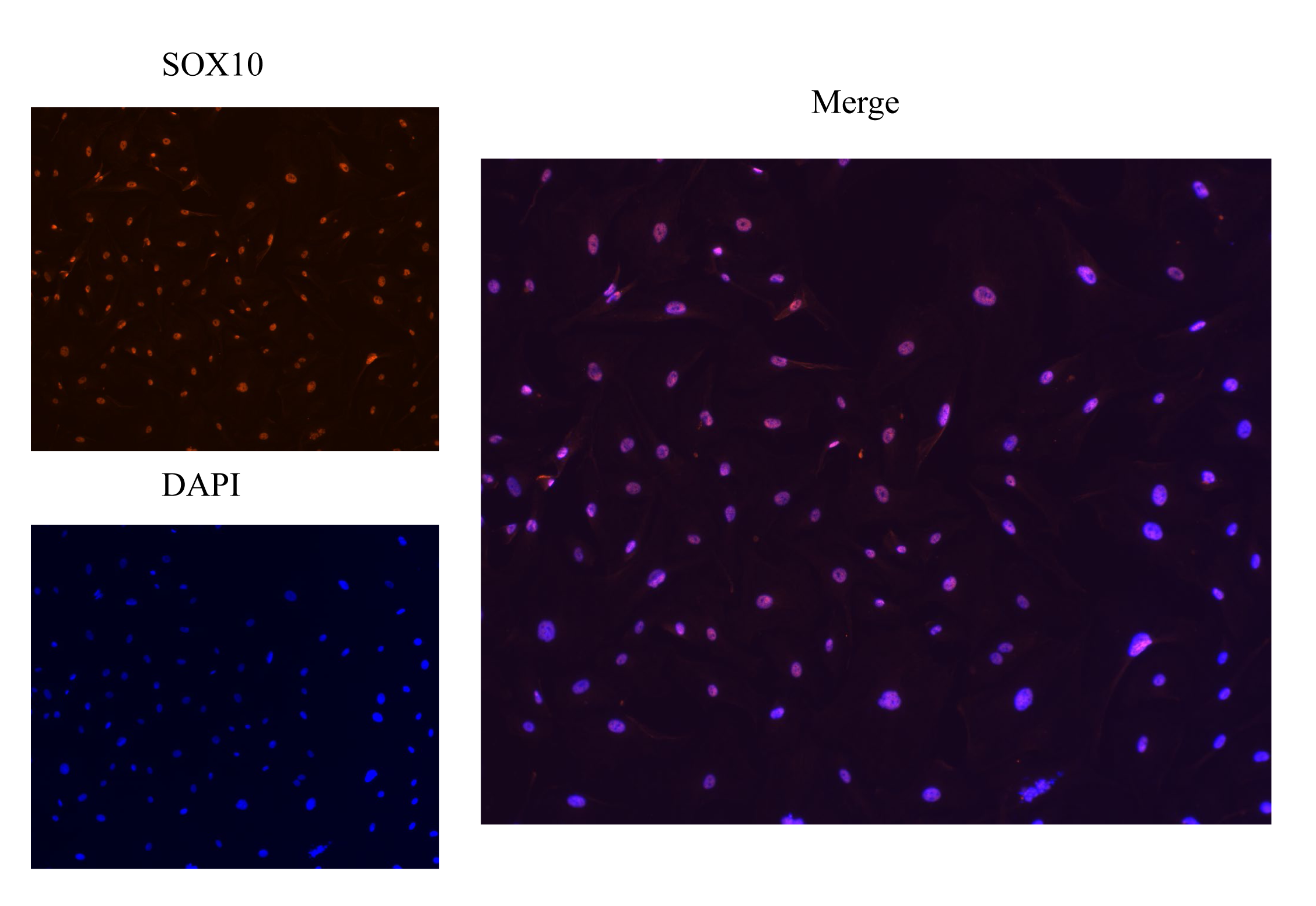

Supplement: Supplementary file 1 [file curroncol-32-00569-s001.zip › sfig1.tif]
